# Dual-source computed tomography for evaluating coronary stenosis and left ventricular function

**DOI:** 10.3892/etm.2013.1253

**Published:** 2013-08-06

**Authors:** CHUNFENG HU, JIE WANG, KAI XU, YINGYING YUAN, XIULING WANG, LIXIANG XIE, SHAODONG LI

**Affiliations:** 1 Department of Radiology, The First Affiliated Hospital of Nanjing Medical University, Nanjing, Jiangsu 210029;; 2 Department of Radiology, The Affiliated Hospital of Xuzhou Medical College, Xuzhou, Jiangsu 221006, P.R. China

**Keywords:** dual-source computed tomography, left ventricular function, echocardiography, coronary stenosis

## Abstract

This study aimed to evaluate the correlation between coronary stenosis and left ventricular function using dual-source computed tomography (DSCT). DSCT coronary angiography (CAG) was performed on 66 patients with coronary disease and 36 healthy volunteers. The degree of coronary stenosis, end-diastolic volume (EDV), end-systolic volume (ESV), stroke volume (SV), ejection fraction (EF) and myocardial mass (MM) were measured for the left ventricle. These values were compared with the results obtained by echocardiography (ECHO) and selective CAG, which were both adopted as controls. The diagnoses of coronary stenosis based on DSCT CAG and those based on selective CAG were not significantly different (P>0.05). Similarly, the values of EDV, ESV, SV or EV measured by DSCT CAG were not significantly different from thoses obtained by ECHO (P>0.05). However, significant differences were observed in the ESV, EF and SV of the severe stenosis group compared with the moderate and mild stenosis groups (both P<0.05). The values of EDV and MM significantly varied between the mild, moderate and severe stenosis groups (P<0.05). DSCT CAG is a highly accurate and highly reproducible method for evaluating the preliminary changes in cardiac function based on the variations of coronary stenosis. Significant changes were detected in the EDV and MM of the moderate stenosis group and in all parameters of the severe stenosis group.

## Introduction

Clinical imaging uses a variety of methods for the evaluation of coronary arteries and ventricular function; these techniques are categorized as either invasive or non-invasive and include X-ray cardioangiography, transesophageal echocardiography (ECHO), transthoracic ECHO, cardiac magnetic resonance imaging, cardiovascular radionuclide imaging and cardiovascular computed tomography (CT) imaging. Compared with the traditional single-source multi-slice spiral CT, dual-source CT (DSCT) has a faster scanning rate and a higher time resolution of 83 msec. Therefore, coronary imaging using DSCT is less susceptible to interference from the heart rate and expands the clinical applications of CT in coronary imaging. DSCT is relatively consistent in terms of accurately diagnosing coronary lesions when used with coronary arteriography (also known as coronary angiography, CAG). The imaging quality is susceptible to multiple factors, including heart rate irregularities such as tachycardia and arrhythmia, artifacts due to respiration and body movement, parameters in scanning and reconstruction, selected trigger points in the monitored layer, chosen reconstruction phases, adopted injection velocity, dosage of the contrast and options of pitch (1–4). Studies on the applications of DSCT for evaluating cardiac morphology and function have been considered noteworthy. Despite the possibility of overestimation or underestimation, the left heart function evaluated by DSCT appears to be reliable regardless of its inability to prove significant differences in comparative studies, as compared with ultrasound or MRI as the controls (5–7). Coronary stenosis leads to myocardial ischemia or infarction, thereby negatively influencing cardiac motor function. Different degrees of coronary stenoses differ in the extent of their effect on cardiac function. The evaluation of altered cardiac function based on the extent of coronary stenosis is of great importance in clinical diagnosis, treatment, and prognosis. Only a few studies on this topic have been conducted or are currently ongoing. The present study aimed to determine the accuracy of DSCT for evaluating coronary stenosis and cardiac function (without the intake of heart rate-reducing agents). Selective CAG and transthoracic ECHO were used as controls to determine the correlation between various degrees of stenosis and the altered left ventricular (LV) function, as well as the significance of DSCT in clinical applications.

## Materials and methods

### Clinical data

Data were collected from 66 patients with coronary heart disease, which included 40 males and 26 females aged 41 to 88 years old (mean, 62.7), with heart rates between 65 and 120 bpm as well as normal rhythm. The patients enrolled in the study underwent DSCT CAG between January 2012 and January 2013. A diagnosis was established for every patient based on their history, physical examination, electrocardiography (ECG), serum biochemistry and myocardial enzyme levels. Patients with diseases such as rheumatic heart disease were excluded. The patients underwent CAG and ECHO within one week. This study was conducted in accordance with the Declaration of Helsinki. This study was conducted with approval from the Ethics Committee of the First Affiliated Hospital of Nanjing Medical University, Nanjing, China. Written informed consent was obtained from all participants.

The control group included 36 healthy volunteers who underwent DSCT CAG. This group included 21 males and 15 females aged 16 to 74 years-old (mean, 49.3), with heart rates of 60 to 110 bpm and normal rhythm. The volunteers were healthy; those with a predisposition to any organic diseases were excluded. No abnormalities were observed during the detailed inquiry of patient history, physical examination, ECG, ECHO, serum biochemistry or results of the examination of hepatic or renal function.

### Scanning methods and data collection

Retrospective ECG-gated scanning was performed using a DSCT machine (Somatom Definition; Siemens, Munich, Germany). Prior to the scanning, the participants were prohibited from taking heart rate-reducing agents, such as Betaloc. A scout view was obtained for a patient holding his breath in a supine position, while scanning from 1 cm inferior to the tracheal carina to 1.5 cm inferior to the inferior border of the heart. A plain scan was initially generated prior to the injection of the nonionic contrast agent iohexol (350 mg I/ml) via the ulnar vein at a dosage of 80–100 ml at a rate of 4.5–5.5 ml/s. A contrast-triggered enhanced scanning was then performed with the tracing level at the root of the ascending aorta and the trigger threshold at 90–100 HU. The scan was launched with a 6 sec delay and performed for 5 to 12 sec. Following injection of the contrast agent, 30 ml 0.9% normal saline was injected at the same rate. The following scanning parameters were used: detector dimensions, 2×32×0.6 mm; layer thickness, 2×64×0.6 mm; rack rotation time, 330 msec; heart rate-dependent pitch, 0.2–0.5; tube current time, 400 mAsec/cycle; and tube voltage, 120 kV.

Off-line reconstruction was performed for the raw material after the scanning was finished, with a 1 mm-thick reconstruction layer, a 1 mm interval, a 10% R-R interval, and a convolution function value of B26f. All-phase reconstruction at 0 to 95% was adopted to obtain images of the 20 cardiac cycles, which were transferred to the workstation for analysis by Syngo Circulation (Siemens). The short-axis, four-chamber, and two-chamber views in each cycle were observed in cineloop to define the ends of the diastole and systole. The LVA software (Siemens) was used to analyze the cardiac function and delineate the endocardium and epicardium, by covering the entire area from the outflow tract to the left ventricle; the papillary muscles were irregular and small, and therefore ignored. The end-diastolic volume (EDV), endsystolic volume (ESV), stroke volume (SV), ejection fraction (EF) and myocardial mass (MM) of the left ventricle were automatically calculated based on the Simpson’s rule for numerical integration. The mean of triplicate measurements was recorded. The data included in this study were obtained by one physician proficient in the use of the software.

### Analysis of coronary stenosis shown by angiography

The degree of stenosis was qualitatively evaluated using the area method. The stenoses were categorized into three groups based on the routine standards commonly used in clinical practice, namely, the mild group with <50% stenosis, the moderate group stenosis with between 50 and 75% stenosis, and the severe group with ≥75% stenosis (8). The stenoses in multiple branches were categorized according to the narrowest branch.

### ECHO analysis

Two-dimensional ECHO was performed using a Color Doppler intelligent diagnostic system (Philips IE 33, Amsterdam, The Netherlands). The examinations were performed by one senior physician.

### Statistical analysis

The data were expressed as mean ± standard deviation. A paired χ^2^ test was performed to compare the coronary stenosis evaluated by CAG and DSCT CAG. The two-sample Student’s t-test and the Pearson correlation analysis were used to correlate the cardiac function based on DSCT against the ECHO findings. The two-sample Student’s t-test and q-test were used to analyze the effect of different degrees of coronary stenosis on cardiac function. P<0.05 was considered to indicate a statistically significant result.

## Results

### Coronary stenosis evaluated by DSCT

**Figure 1.**
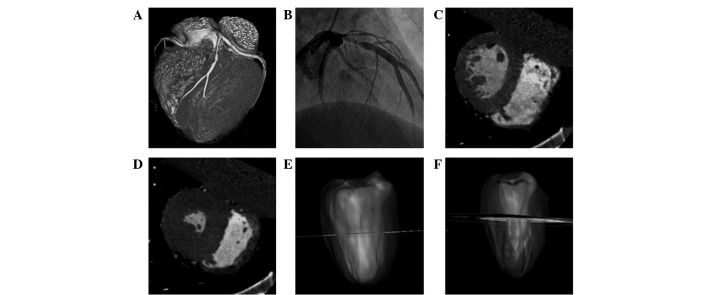
A 59-year-old male with recurrent chest stuffiness in the chest for 1 month. (A) CT-VR; (B) DSA-CAG, a moderate stenosis in the proximal end of the LAD, showing the consistency between the two approaches; (C) short-axis position of the left ventricle at the end of diastasis; (D) short-axis position of the left ventricle at the end of systole; (E) VR of the ventricular volume at the end of diastasis based on cardiac function analysis; (F) VR of ventricular volume at the end of systole. CT-VR, computed tomography-volume render; DSA-CAG, digital subtraction angiography-coronary angiography; LAD, left anterior descending artery; VR, volume render.

Out of the 330 assessable arteries, 211 exhibited stenosis based on the DSCT results [right coronary artery (RCA), left anterior descending artery (LAD), diagonal branch (Diag), left circumflex coronary artery (LCX), left obtuse marginal branch (LOM)] of 66 patients, out of which 196 were confirmed by selective CAG. Only 12 instances of selective CAG-identified stenoses were not detected by DSCT CAG (Table I). The paired χ^2^ test (McNemar test) with P=0.701 (P>0.05) and κ coefficient test (kappa test) with κ=0.824 (κ≥0.7; P=0.000) suggested a high level of consistency between the DSCT CAG and selective CAG results. No significant differences in the capacity to diagnose coronary stenosis were observed between the two approaches (Fig. 1A and B).

### Comparison of LV function as evaluated by DSCT and ECHO

The results showed that the EDV, SV and EF of the left ventricle in the disease and control groups were slightly higher, while ESV was slightly lower when measured by DSCT, as compared with those measured by ECHO. However, no significant differences were observed between the parameters of cardiac function measured by the two methods (P>0.05; Tables II and III).

### Association between the degree of coronary stenosis and the parameters of LV function

There were 36 individuals in the control group, and 8, 22 and 36 patients in the mild, moderate and severe stenosis groups, respectively. The q-test of paired comparisons revealed significant differences in the ESV, EF and SV in the severe group, as compared with those of the mild or the moderate groups (both P<0.05). However, these parameters were not significantly different between the mild and the moderate groups (P>0.05). Significant differences were observed in EDV and MM among the three stenosis groups (P<0.05). No significant differences were demonstrated among the parameters of the control group, as compared with those of the mild stenosis group (P>0.05; Table IV).

## Discussion

The use of CAG with spiral CT has become an important invasive approach for coronary imaging. In addition, the parameters of ventricular function may be obtained while angiography is conducted and thus provide more informative data for clinical practice. The dosage of heart rate-reducing agents prior to the examination may interfere with the results (9) Therefore, the results of the DSCT CAG performed without artificial control of the heart rate may overestimate the actual values. The extent of coronary stenosis similarly influences the alteration of LV function to a certain degree.

Previous studies (10,11) have revealed the high sensitivity, specificity and accuracy of DSCT performed under stable heart rate conditions without obvious variability or arrhythmia, in diagnosing significant stenosis in coronary arteries. Similarly, our results from patients who did not take heart rate-reducing agents suggested that DSCT CAG is capable of consistently diagnosing coronary stenosis without significant differences. However, multiple factors may affect CAG. Incorrect or incomplete diagnoses of the patients may be correlated with the flowing velocity of the contrast, twisted courses of blood vessels and the calcification in the vascular wall. Certain studies (12–14) have suggested that the tendency of multi-slice spiral CT to overestimate coronary stenosis is correlated with the spatial resolution being used, particularly the effect of the partial volume effect on the calcified vessel wall. Despite its inability to provide hemodynamic information, CT may rule out the presence of obvious coronary stenosis, therefore, this method has important applications in clinical practice. Therefore, a close-up view from multiple angles with numerous aspects should be used while analyzing CAG to minimize errors or missed diagnoses.

By distinguishing the defined borders of the endocardium and epicardium, DSCT may reveal the anatomical structure of the heart more clearly than ultrasound scan. Due to its large capacity for raw data and a powerful post-processing software, DSCT is capable of semi-automatically delineating the ventricular contours and enabling the repeated playback of three-dimensional movies that simultaneously show multiple positions of the heart (Fig. 1C–F), thereby indicating its high reproducibility (15). ECHO is the most frequently used approach for clinically evaluating cardiac function and it was used as the control. The results of DSCT and ECHO were highly consistent; their values for EDV, ESV, SV and EF were not significantly different. However, the parameters evaluated by DSCT (with the exception of ESV) were slightly higher than those by ECHO. Previous studies suggested that overestimation or underestimation exists in both MDCT and DSCT when ECHO is adopted as the control (6,16,17). These inconsistencies are due to the lack of similar temporal resolution among the different techniques. However, the differences have no statistical significance, which shows that these techniques are of great clinical importance. Systematic underestimation exists in various parameters of cardiac function evaluated by MRI, while the underestimation or overestimation occurs in results evaluated by the 64-layer spiral CT due to its dependence upon the heart rate. A comparison of the two approaches indicated that DSCT appeared to have the least number of errors (18). In addition, no heart rate-reducing agent was required by DSCT, thereby eliminating the influence of artificial factors on cardiac function and making the results more accurate.

Coronary stenosis leads to myocardial ischemia. The blood flow reserve for coronary vessels gradually decreases with the increasing severity of coronary stenosis, and resting ventricular ataxia occurs at this stage. This proceeds to altered cardiac functions, including weakened myocardial contractility, enlarged heart and decreased compliance, as well as congestion in the left ventricle and the left atria. The increased EDV and decreased EF reflect the gradual loss of compensation on the left side of the heart, as well as predict the onset of left-sided heart failure and poor prognosis. Myocardial ischemia and anoxia may cause myocardial hypertrophy and thickened ventricular walls, thereby increasing MM but decreasing myocardial contractility. In clinical practice, the evaluation of varying cardiac functions according to the degree of coronary stenosis is of great importance. Along with the increasing number of coronary branches with lesions and the aggravating stenosis, cardiac function gradually worsens. In this study, patients were categorized into three groups by evaluating the coronary stenosis, which is frequently used in clinical practice. No significant variation was observed in the parameters in the mild group compared with those of the controls. Similarly, no significant changes were observed in the ESV, SV or EF in the moderate stenosis group. However, significant changes were noted in the severe stenosis group. Myocardial compensation continued to be achieved in certain cases of mild or moderate stenosis of the coronary arteries. The enhanced myocardial contractibility may aid maintenance of systemic circulation and avoid a significant reduction of the EF, which may occasionally undergo a compensatory increase. A significant reduction in the EF occurred with the decompensation of cardiac function in the severe stenosis group. The decreasing EF was closely correlated with the incidence and mortality of acute myocardial infarction. EDV and MM were significantly increased in the moderate stenosis group, and increased further in the severe group relative to the moderate group. Therefore, EDV and MM are likely more sensitive than EF in terms of reflecting the variation of cardiac function. Along with the increasing EDV that occurs with ventricular wall tension, myocardial oxygen consumption was further increased with aggravated myocardial ischemia. Simultaneously, the MM may gradually increase and further aggravate cardiac contractibility, thereby suggesting that severe myocardial ischemia damages LV systolic functions.

This study is primarily limited by the use of only the mild, moderate and severe categories in the interest of simplification and pragmatism. Our results would have been more meaningful if stenosis sites at the trunk or branch, as well as the lesions in single or multiple branches were considered. Our study is further limited by the size of the samples used. Therefore, further studies are required to investigate patients with different types of arrhythmias. Despite its high temporal and spatial resolution, DSCT has a limited capacity to accurately identify stenosis due to motion and halo artifacts induced by arrhythmias and calcification, respectively. DSCT is similarly challenged by controversies concerning ionizing radiation (19,20). Thus, future studies are required to focus on the realization of scanning with reduced radiation.

None of the previously available data indicate that heart CT scans have been used to evaluate cardiac function alone. However, data with regard to cardiac function may be utilized during CAG to obtain additional information (12). Our results suggest that the degree of coronary stenosis may be used to evaluate variations in cardiac function. Significant changes to the EDV and MM in the left ventricle of the moderate stenosis group, as well as those of all the parameters in the severe group, may aid in the diagnosis, early intervention and prognosis of stenosis. Therefore, acute coronary events and heart failure may be prevented. DSCT during one examination is able to accomplish the simultaneous observation of two parameters, including the morphology of coronary vessels and the biology of EFs. Therefore, DSCT is the fastest method for non-invasive cardiovascular examination, with clinical applications of great significance.

## Figures and Tables

**Table I.** Comparative analysis between coronary artery DSCT-CTA and the selective CAG control.

Coronary artery DSCT-CTA	CAG		Total (segment)
	Positive (segment)	Negative (segment)	
Positive (segment)	196	15	211
Negative (segment)	12	107	119
Total (segment)	208	122	330

DSCT, dual-source computed tomography; CTA, computed tomography angiography; CAG, coronary angiography.

**Table II.** Comparison of left ventricular function between DSCT and ECHO in the control group.

Parameters of left ventricular function	DSCT	ECHO	r-value	P-value
EDV (ml)	100.78±13.78	97.61±13.32	0.81	0.92
ESV (ml)	31.56±7.44	31.61±6.99	0.90	0.91
SV (ml)	69.22±10.96	66.00±9.88	0.71	0.96
EF (%)	68.67±5.88	67.69±5.19	0.83	0.65

DSCT, dual-source computed tomography; ECHO, echocardiography; EDV, end-diastolic volume; ESV, end-systolic volume; SV, stroke volume; EF, ejection fraction.

**Table III.** Comparison of determined parameters of left heart function by DSCT with those by ECHO in the stenosis group.

Parameters of left ventricular function	DSCT	ECHO	r-value	P-value
EDV (ml)	124.85±50.81	121.20±48.15	0.76	0.91
ESV (ml)	51.91±38.96	52.25±36.02	0.91	0.88
SV (ml)	72.02±25.87	68.95±24.43	0.72	0.89
EF (%)	59.72±13.68	58.45±11.81	0.83	0.69

DSCT, dual-source computed tomography; ECHO, echocardiography; EDV, end-diastolic volume; ESV, end-systolic volume; SV, stroke volume; EF, ejection fraction.

**Table IV.** Correlation between the degree of coronary stenosis and parameters of left ventricular function.

Parameters of left ventricular function	Control	Mild stenosis	Moderate stenosis	Severe stenosis	P-value
EDV (ml)	100.78±13.78	104.56±20.21	115.43±23.66	138.34±36.14	<0.05
ESV (ml)	31.56±7.44	35.42±20.53	38.54±21.64	59.21±43.56	>0.05^a^
SV (ml)	69.22±10.96	72.75±10.44	66.84±15.76	41.43±13.28	>0.05^a^
EF (%)	68.67±5.88	67.29±11.34	66.37±12.98	31.76±8.97	>0.05^a^
MM (g)	97.60±17.64	107.45±12.74	136.86±8.32	155.46±34.07	<0.05

No significance difference was identified in all parameters between the normal group and the mild stenosis group;

a No statistical significance in the difference between the mild and moderate stenosis groups. EDV, end-diastolic volume; ESV, end-systolic volume; SV, stroke volume; EF, ejection fraction; MM, myocardial mass.
